# Supplementary material for: Secretion of protein disulphide isomerase AGR2 confers tumorigenic properties
Source: eLife. 2016 May 30;5:e13887. doi: 10.7554/eLife.13887 (PMC4940162; doi:10.7554/eLife.13887)
Supplement: Supplementary file 1. — (A) AGR2 staining and clinicopathological characteristics of human lung cancer tissues samples analysed. (B) Gene Table of EMT Signaling pathway. DOI: http://dx.doi.org/10.7554/eLife.13887.017 [file elife-13887-supp1.doc]

**Supplementary file 1A - AGR2 staining and clinicopathological characteristics of human lung cancer tissues samples analysed.**

| ***Characteristics*** | ***Case, n*** | ***Age*** | | ***Sex*** | | ***AGR2 Intensity*** | | |
| --- | --- | --- | --- | --- | --- | --- | --- | --- |
|  |  | ***<45*** | ***>45*** | ***M*** | ***F*** | ***Low*** | ***Medium*** | ***High*** |
| Lung Cancer | 34 | 2 | 32 | 26 | 8 | 7 | 11 | 16 |
|  |  |  |  |  |  |  |  |  |
| Large cells | 3 | 0 | 3 | 1 | 2 | 0 | 2 | 1 |
| Squamous lung carcinoma | 14 | 1 | 13 | 13 | 1 | 5 | 5 | 4 |
| Adenocarcinoma | 17 | 1 | 16 | 12 | 5 | 2 | 3 | 12 |
| Stage |  |  |  |  |  |  |  |  |
| IIA | 3 | 0 | 3 | 2 | 1 | 0 | 1 | 2 |
| IIB | 7 | 0 | 7 | 5 | 2 | 4 | 2 | 1 |
| IIIA | 20 | 1 | 19 | 16 | 4 | 3 | 7 | 10 |
| IIIB | 4 | 1 | 3 | 3 | 1 | 0 | 1 | 3 |

**Supplementary file 1B - Gene Table of EMT Signalling pathway.**

| ***Position*** | ***Unigene*** | ***GeneBank*** | ***Symbol*** | ***Description*** | ***Gene name*** |
| --- | --- | --- | --- | --- | --- |
| A01 | Hs.502756 | NM_024060 | AHNAK | AHNAK nucleoprotein | AHNAKRS |
| A02 | Hs.525622 | NM_005163 | AKT1 | V-akt murine thymoma  viral oncogene homolog 1 | AKT/CWS6/PKB/PKB-ALPHA/PRKBA/RAC/RAC-ALPHA |
| A03 | Hs.1274 | NM_006129 | BMP1 | Bone morphogenetic  protein 1 | OI13/PCOLC/PCP/PCP2/TLD |
| A04 | Hs.73853 | NM_001200 | BMP2 | Bone morphogenetic  protein 2 | BDA2/BMP2A |
| A05 | Hs.473163 | NM_001719 | BMP7 | Bone morphogenetic  protein 7 | OP-1 |
| A06 | Hs.490203 | NM_004342 | CALD1 | Caldesmon 1 | CDM/H-CAD/HCAD/L-CAD/LCAD/NAG22 |
| A07 | Hs.731383 | NM_018584 | CAMK2N1 | Calcium/calmodulin-  dependent protein kinase  II inhibitor 1 | PRO1489/RP11-401M16.1 |
| A08 | Hs.603096 | NM_001233 | CAV2 | Caveolin 2 | CAV |
| A09 | Hs.461086 | NM_004360 | CDH1 | Cadherin 1, type 1, E-cadherin (epithelial) | Arc-1/CD324/CDHE/ECAD/LCAM/UVO |
| A10 | Hs.606106 | NM_001792 | CDH2 | Cadherin 2, type 1, N-cadherin (neuronal) | CD325/CDHN/CDw325/NCAD |
| A11 | Hs.489142 | NM_000089 | COL1A2 | Collagen, type I, alpha 2 | OI4 |
| A12 | Hs.443625 | NM_000090 | COL3A1 | Collagen, type III, alpha 1 | EDS4A |
| B01 | Hs.445827 | NM_000393 | COL5A2 | Collagen, type V, alpha 2 | - |
| B02 | Hs.476018 | NM_001904 | CTNNB1 | Catenin (cadherin-associated protein), beta 1, 88kDa | CTNNB/MRD19/armadillo |
| B03 | Hs.95612 | NM_004949 | DSC2 | Desmocollin 2 | ARVD11/CDHF2/DG2/DGII/III/DSC3 |
| B04 | Hs.519873 | NM_004415 | DSP | Desmoplakin | DP/DPI/DPII |
| B05 | Hs.605083 | NM_005228 | EGFR | Epidermal growth factor receptor | ERBB/ERBB1/HER1/PIG61/mENA |
| B06 | Hs.622058 | NM_001982 | ERBB3 | V-erb-b2 erythroblastic leukemia viral oncogene homolog 3 (avian) | ErbB-3/HER3/LCCS2/MDA-BF-1/c-erbB-3/c-erbB3/erbB3-S/p180-ErbB3/p45-sErbB3/p85-sErbB3 |
| B07 | Hs.208124 | NM_000125 | ESR1 | Estrogen receptor 1 | ER/ESR/ESRA/ESTRR/Era/NR3A1 |
| B08 | Hs.517293 | NM_016946 | F11R | F11 receptor | CD321/JAM/JAM1/JAMA/JCAM/KAT/PAM-1 |
| B09 | Hs.1690 | NM_005130 | FGFBP1 | Fibroblast growth factor binding protein 1 | FGF-BP/FGF-BP1/FGFBP/FGFBP-1/HBP17 |
| B10 | Hs.203717 | NM_002026 | FN1 | Fibronectin 1 | CIG/ED-B/FINC/FN/FNZ/GFND/GFND2/LETS/MSF |
| B11 | Hs.436448 | NM_005251 | FOXC2 | Forkhead box C2 (MFH-1, mesenchyme forkhead 1) | FKHL14/LD/MFH-1/MFH1 |
| B12 | Hs.173859 | NM_003507 | FZD7 | Frizzled family receptor 7 | FzE3 |
| C01 | Hs.83381 | NM_004126 | GNG11 | Guanine nucleotide binding protein (G protein), gamma 11 | GNGT11 |
| C02 | Hs.440438 | NM_173849 | GSC | Goosecoid homeobox | - |
| C03 | Hs.445733 | NM_002093 | GSK3B | Glycogen synthase kinase 3 beta | - |
| C04 | Hs.462998 | NM_001552 | IGFBP4 | Insulin-like growth factor binding protein 4 | BP-4/HT29-IGFBP/IBP4/IGFBP-4 |
| C05 | Hs.81134 | NM_000577 | IL1RN | Interleukin 1 receptor antagonist | DIRA/ICIL-1RA/IL-1RN/IL-1ra/IL-1ra3/IL1F3/IL1RA/IRAP/MVCD4 |
| C06 | Hs.706355 | NM_004517 | ILK | Integrin-linked kinase | HEL-S-28/ILK-1/ILK-2/P59/p59ILK |
| C07 | Hs.505654 | NM_002205 | ITGA5 | Integrin, alpha 5 (fibronectin receptor, alpha polypeptide) | CD49e/FNRA/VLA5A |
| C08 | Hs.436873 | NM_002210 | ITGAV | Integrin, alpha V (vitronectin receptor, alpha polypeptide, antigen CD51) | CD51/MSK8/VNRA/VTNR |
| C09 | Hs.643813 | NM_002211 | ITGB1 | Integrin, beta 1 (fibronectin receptor, beta polypeptide, antigen CD29 includes MDF2, MSK12) | CD29/FNRB/GPIIA/MDF2/MSK12/VLA-BETA/VLAB |
| C10 | Hs.626544 | NM_000214 | JAG1 | Jagged 1 | AGS/AHD/AWS/CD339/HJ1/JAGL1 |
| C11 | Hs.654380 | NM_000526 | KRT14 | Keratin 14 | CK14/EBS3/EBS4/K14/NFJ |
| C12 | Hs.654568 | NM_002276 | KRT19 | Keratin 19 | CK19/K19/K1CS |
| D01 | Hs.670221 | NM_005556 | KRT7 | Keratin 7 | CK7/K2C7/K7/SCL |
| D02 | Hs.335079 | NM_005909 | MAP1B | Microtubule-associated protein 1B | FUTSCH/MAP5 |
| D03 | Hs.513617 | NM_004530 | MMP2 | Matrix metallopeptidase 2 (gelatinase A, 72kDa gelatinase, 72kDa type IV collagenase) | CLG4/CLG4A/MMP-II/MONA/TBE-1 |
| D04 | Hs.375129 | NM_002422 | MMP3 | Matrix metallopeptidase 3 (stromelysin 1, progelatinase) | CHDS6/MMP-3/SL-1/STMY/STMY1/STR1 |
| D05 | Hs.297413 | NM_004994 | MMP9 | Matrix metallopeptidase 9 (gelatinase B, 92kDa gelatinase, 92kDa type IV collagenase) | CLG4B/GELB/MANDP2/MMP-9 |
| D06 | Hs.87752 | NM_002444 | MSN | Moesin | HEL70 |
| D07 | Hs.517973 | NM_002447 | MST1R | Macrophage stimulating 1 receptor (c-met-related tyrosine kinase) | CD136/CDw136/PTK8/RON |
| D08 | Hs.370414 | NM_018055 | NODAL | Nodal homolog (mouse) | HTX5 |
| D09 | Hs.495473 | NM_017617 | NOTCH1 | Notch 1 | TAN1/hN1 |
| D10 | Hs.533657 | NM_015901 | NUDT13 | Nudix (nucleoside diphosphate linked moiety X)-type motif 13 | - |
| D11 | Hs.592605 | NM_002538 | OCLN | Occludin | BLCPMG |
| D12 | Hs.509067 | NM_002609 | PDGFRB | Platelet-derived growth factor receptor, beta polypeptide | CD140B/IBGC4/IMF1/JTK12/PDGFR/PDGFR-1/PDGFR1 |
| E01 | Hs.170473 | NM_016445 | PLEK2 | Pleckstrin 2 | - |
| E02 | Hs.570455 | NM_015704 | DESI1 | PPPDE peptidase domain containing 2 | D15Wsu75e/DESI2/DJ347H13.4/DeSI-1/FAM152B/PPPDE2 |
| E03 | Hs.395482 | NM_005607 | PTK2 | PTK2 protein tyrosine kinase 2 | FADK/FAK/FAK1/FRNK/PPP1R71/p125FAK/pp125FAK |
| E04 | Hs.706850 | NM_003463 | PTP4A1 | Protein tyrosine phosphatase type IVA, member 1 | HH72/PRL-1/PRL1/PTP(CAAX1)/PTPCAAX1 |
| E05 | Hs.413812 | NM_006908 | RAC1 | Ras-related C3 botulinum toxin substrate 1 (rho family, small GTP binding protein Rac1) | Rac-1/TC-25/p21-Rac1 |
| E06 | Hs.78944 | NM_002923 | RGS2 | Regulator of G-protein signaling 2, 24kDa | G0S8 |
| E07 | Hs.713079 | NM_000602 | SERPINE1 | Serpin peptidase inhibitor, clade E (nexin, plasminogen activator inhibitor type 1), member 1 | PAI/PAI-1/PAI1/PLANH1 |
| E08 | Hs.652307 | NM_003616 | GEMIN2 | Survival of motor neuron protein interacting protein 1 | SIP1/SIP1-delta |
| E09 | Hs.705764 | NM_005901 | SMAD2 | SMAD family member 2 | JV18/JV18-1/MADH2/MADR2/hMAD-2/hSMAD2 |
| E10 | Hs.48029 | NM_005985 | SNAI1 | Snail homolog 1 (Drosophila) | SLUGH2/SNA/SNAH/SNAIL/SNAIL1/dJ710H13.1 |
| E11 | Hs.360174 | NM_003068 | SNAI2 | Snail homolog 2 (Drosophila) | SLUG/SLUGH1/SNAIL2/WS2D |
| E12 | Hs.673548 | NM_178310 | SNAI3 | Snail homolog 3 (Drosophila) | SMUC/SNAIL3/ZNF293/Zfp293 |
| F01 | Hs.376984 | NM_006941 | SOX10 | SRY (sex determining region Y)-box 10 | DOM/PCWH/WS2E/WS4/WS4C |
| F02 | Hs.111779 | NM_003118 | SPARC | Secreted protein, acidic, cysteine-rich (osteonectin) | ON |
| F03 | Hs.313 | NM_000582 | SPP1 | Secreted phosphoprotein 1 | BNSP/BSPI/ETA-1/OPN |
| F04 | Hs.463059 | NM_003150 | STAT3 | Signal transducer and activator of transcription 3 (acute-phase response factor) | APRF/HIES |
| F05 | Hs.61635 | NM_012449 | STEAP1 | Six transmembrane epithelial antigen of the prostate 1 | PRSS24/STEAP |
| F06 | Hs.657044 | NM_003200 | TCF3 | Transcription factor 3 (E2A immunoglobulin enhancer binding factors E12/E47) | E2A/E47/ITF1/TCF-3/VDIR/bHLHb21 |
| F07 | Hs.605153 | NM_003199 | TCF4 | Transcription factor 4 | E2-2/ITF-2/ITF2/PTHS/SEF-2/SEF2/SEF2-1/SEF2-1A/SEF2-1B/SEF2-1D/TCF-4/bHLHb19 |
| F08 | Hs.438231 | NM_006528 | TFPI2 | Tissue factor pathway inhibitor 2 | PP5/REF1/TFPI-2 |
| F09 | Hs.645227 | NM_000660 | TGFB1 | Transforming growth factor, beta 1 | CED/DPD1/LAP/TGFB/TGFbeta |
| F10 | Hs.133379 | NM_003238 | TGFB2 | Transforming growth factor, beta 2 | LDS4/TGF-beta2 |
| F11 | Hs.592317 | NM_003239 | TGFB3 | Transforming growth factor, beta 3 | ARVD/ARVD1/RNHF/TGF-beta3 |
| F12 | Hs.522632 | NM_003254 | TIMP1 | TIMP metallopeptidase inhibitor 1 | CLGI/EPA/EPO/HCI/TIMP |
| G01 | Hs.598100 | NM_003692 | TMEFF1 | Transmembrane protein with EGF-like and two follistatin-like domains 1 | C9orf2/CT120.1/H7365/TR-1 |
| G02 | Hs.118552 | NM_178031 | TMEM132A | Transmembrane protein 132A | GBP/HSPA5BP1 |
| G03 | Hs.364544 | NM_014399 | TSPAN13 | Tetraspanin 13 | NET-6/NET6/TM4SF13 |
| G04 | Hs.644998 | NM_000474 | TWIST1 | Twist homolog 1 (Drosophila) | ACS3/BPES2/BPES3/CRS1/SCS/TWIST/bHLHa38 |
| G05 | Hs.643801 | NM_004385 | VCAN | Versican | CSPG2/ERVR/GHAP/PG-M/WGN/WGN1 |
| G06 | Hs.691131 | NM_003380 | VIM | Vimentin | CTRCT30/HEL113 |
| G07 | Hs.662256 | NM_033305 | VPS13A | Vacuolar protein sorting 13 homolog A (S. cerevisiae) | CHAC/CHOREIN |
| G08 | Hs.108219 | NM_004626 | WNT11 | Wingless-type MMTV integration site family, member 11 | HWNT11 |
| G09 | Hs.643085 | NM_003392 | WNT5A | Wingless-type MMTV integration site family, member 5A | hWNT5A |
| G10 | Hs.306051 | NM_032642 | WNT5B | Wingless-type MMTV integration site family, member 5B | - |
| G11 | Hs.124503 | NM_030751 | ZEB1 | Zinc finger E-box binding homeobox 1 | AREB6/BZP/DELTAEF1/FECD6/NIL2A/PPCD3/TCF8/ZFHEP/ZFHX1A |
| G12 | Hs.34871 | NM_014795 | ZEB2 | Zinc finger E-box binding homeobox 2 | HSPC082/SIP-1/SIP1/SMADIP1/ZFHX1B |
| H01 | Hs.520640 | NM_001101 | ACTB | Actin, beta | BRWS1/PS1TP5BP1 |
| H02 | Hs.534255 | NM_004048 | B2M | Beta-2-microglobulin | - |
| H03 | Hs.544577 | NM_002046 | GAPDH | Glyceraldehyde-3-phosphate dehydrogenase | G3PD/GAPD |
| H04 | Hs.412707 | NM_000194 | HPRT1 | Hypoxanthine phosphoribosyltransferase 1 | HGPRT/HPRT |
| H05 | Hs.546285 | NM_001002 | RPLP0 | Ribosomal protein, large, P0 | L10E/LP0/P0/PRLP0/RPP0 |
| H06 | N/A | SA_00105 | HGDC | Human Genomic DNA Contamination | HIGX1A |
| H07 | N/A | SA_00104 | RTC | Reverse Transcription Control | RTC |
| H08 | N/A | SA_00104 | RTC | Reverse Transcription Control | RTC |
| H09 | N/A | SA_00104 | RTC | Reverse Transcription Control | RTC |
| H10 | N/A | SA_00103 | PPC | Positive PCR Control | PPC |
| H11 | N/A | SA_00103 | PPC | Positive PCR Control | PPC |
| H12 | N/A | SA_00103 | PPC | Positive PCR Control | PPC |
